# Supplementary material for: Comparison of Two Distinct Subpopulations of Klebsiella pneumoniae ST16 Co-Occurring in a Single Patient
Source: Microbiol Spectr. 2022 Apr 25;10(3):e02624-21. doi: 10.1128/spectrum.02624-21 (PMC9241866; doi:10.1128/spectrum.02624-21)
Supplement: SUPPLEMENTAL FILE 1 — Supplemental material. Download spectrum.02624-21-s001.pdf, PDF file, 0.2 MB [file spectrum.02624-21-s001.pdf]

Table S1. Primers used for PCR detection

Primers	Sequence (5′ to 3′)	Product（bp）	Reference
bla$_{NDM}$-F	GCAGCTTGTCGGCCATGCGGGC	782	this study
bla$_{NDM}$-R	GGTCGCGAAGCTGAGCACCGCAT		
bla$_{OXA-181}$-F	CCACACATTATCATCAAGTTC	418	a
bla$_{OXA-181}$-R	GTGGGATGGACAGACGCG		
bla$_{CMY-2}$ like-F	GGCGGGTTTACCTCAACGGC	943	b
bla$_{CMY-2}$ like-R	TGCTGCTGACAGCCTCTTTCTC		
bla$_{OXA-1}$-F	TATCAACTTCGCTATTTTTTTA	807	c
bla$_{OXA-1}$-R	TTTAGTGTGTTTAGAATGGTGA		
tet(A)-F	CCACGCTCCGTTCTTCG	635	this study
tet(A)-R	ATGCCCACCCGTTCCAC		
catB3-F	AGTATTACCTGCTTTTT	504	this study
catB3-R	GTAGTTTCTGCTCTATC		
aac(6')-Ib-cr-F	ATGACTGAGCATGACCTT	525	c
aac(6')-Ib-cr-R	GAAGGGTTAGGCATCACT		
ompK36-F	TATTTCCCTGACCATTT	1418	this study
ompK36-R	TCGAGGCTCCTCTTACC		
acrR-F	ACGTAACCTCTGTAAAGTCAT	677	d
acrR-R	TTAAGCTGACAAGCTCTCCG		

a. Balm MND, Ngan G, Jureen R, Lin RTP, Teo JWP. 2013. OXA-181-producing *Klebsiella pneumoniae* establishing in Singapore. *BMC Infect Dis* 13:58.

b. Lorme F, Maataoui N, Rondinaud E, Esposito-Farese M, Clermont O, Ruppe E, Arlet G, Genel N, Matheron S, Andremont A, Armand-Lefevre L, Grp V-RS. 2018. Acquisition of plasmid-mediated cephalosporinase producing *Enterobacteriaceae* after a travel to the tropics. *PLoS One* 13:e0206909.

c. Dahmen S, Bettaieb D, Mansour W, Boujaafar N, Bouallegue O, Arlet G. 2010. Characterization and Molecular Epidemiology of Extended-Spectrum beta-Lactamases in Clinical Isolates of *Enterobacteriaceae* in a Tunisian University Hospital. *Microb Drug Resist* 16:163-170.

d. Li RL, Han YJ, Zhou YH, Du ZM, Wu H, Wang J, Chen Y. 2017. Tigecycline Susceptibility and Molecular Resistance Mechanisms Among Clinical *Klebsiella pneumoniae* Strains Isolated During Non-Tigecycline Treatment. *Microb Drug Resist* 23:139-146.

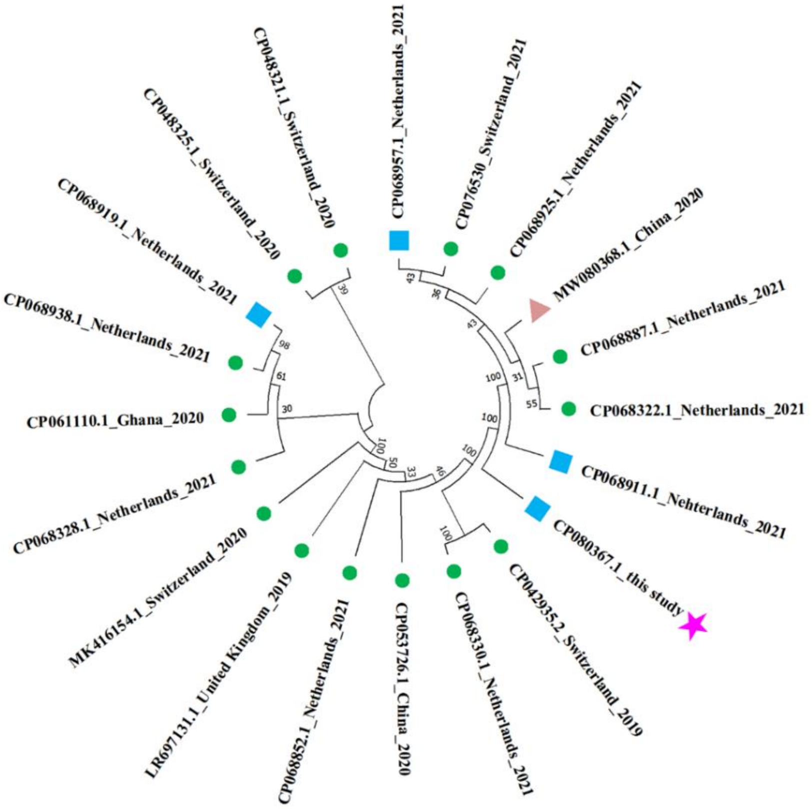

Fig S1. Phylogenetic neighbor-joining tree of results of plasmid comparison with BLAST in the GenBank database (accessed 1st December 2021), together with pOXA181-191773(marked with star). *E. coli* strains were marked with a green circle, *K. pneumoniae* strains were marked with a blue square, and *Morganella morganii* was marked with a pink triangle.

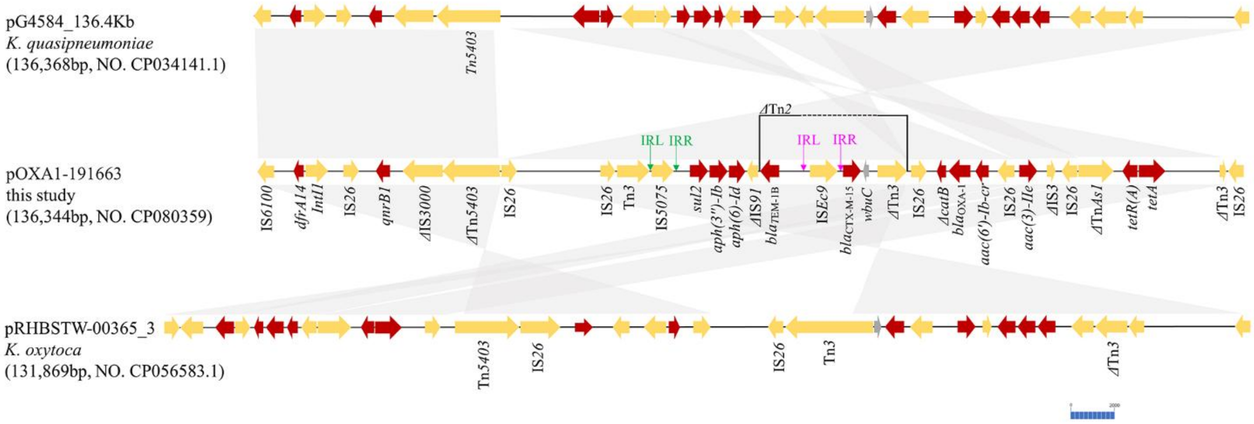

Fig S2. Schematic diagram comparing the multi-resistant region of pOXA1-191663 with those of pG4584_136.4Kb from *K. quasipneumoniae* and pRHBSTW-00365_3 from *K. oxytoca*. Arrowhead indicated the direction of transcription, with red for resistance genes, yellow for transposon or insertion sequence. *wbuc*, encoding cupin fold metalloprotein. Light-gray shading represents the homology region (>99% nucleotide identity). Δ, denotes deletion or truncation. IRL, left inverted repeat; IRR, right inverted repeat. A Tn*2* variant was marked on the diagram.
